# Role of Gut Microbes in Hypertension: A Systematic Review of Literature

**DOI:** 10.3400/avd.ra.24-00121

**Published:** 2025-11-05

**Authors:** Swapnil S Garde, Somesh Garde

**Affiliations:** 1 Interventional Cardiology Services, Sagar Multi-specialty Hospital, Bhopal, India; 2 Critical Care Medicine, Sagar Multi-specialty Hospital, Bhopal, India

**Keywords:** hypertension, gut microbiota, cardiovascular disease, dysbiosis, blood pressure, endothelial function, gut barrier, probiotics, microbial interventions

## Abstract

**Objectives:**

Hypertension is a metabolic disorder affecting a significant proportion of the global population. Growing evidence suggests the contribution of gut microbiota to blood pressure homeostasis and the effectiveness of antihypertensive interventions. This systematic review evaluates the role of gut microbiota in hypertension and identifies microbial taxa contributing to or alleviating the condition.

**Methods:**

A systematic search was conducted in PubMed and Cochrane databases for non-randomized studies, randomized controlled trials, and registry studies published in English. Studies were classified according to microbial taxa involved in the improvement or worsening of hypertension.

**Results:**

According to the inclusion criteria, 19 studies were included. Some bacterial genera, such as *Lactobacillus paracasei*, *Akkermansia*, and *Veillonella*, had potential protective effects against hypertension by regulating blood pressure through dietary interactions and microbial metabolites. On the other hand, *Klebsiella* sp., *Streptococcus* sp., and *Parabacteroides merdae* were more abundant in hypertensive patients and were involved in dysbiosis and inflammation. The fungal taxa *Malassezia* and *Mortierella* were also involved in the pathogenesis of hypertension.

**Conclusions:**

Gut microbiota composition may play crucial roles in hypertension, with certain taxa potentially contributing to or alleviating the condition. Modulating gut microbes through probiotics and diet may offer new therapeutic approaches.

## Introduction

Hypertension (HTN) is a metabolic disorder affecting a significant proportion of the global population. It occurs due to the superimposition of genetic and environmental factors.^1–3)^ Previous studies have focused mainly on vascular remodeling and central regulation. Environmental factors, especially the internal ecosystem of the human body, such as the gut microbiome, are often neglected.^4)^ Investigations have observed gut microbiota as an essential modulator of environmental factors that play key roles in disease development and progression, and that mediate pharmacological interventions.^5–9)^

Increasing studies in humans have demonstrated that an imbalance of gut microbiota plays a key role in the occurrence and development of HTN.^10,11)^ Conversely, HTN can also significantly impact the structure and composition of the gut microbiota.^12,13)^ While reviews conducted previously have discussed the influence of gut microbiota on HTN, comprehensive reviews highlighting the gut microbes that contribute to or alleviate HTN are lacking. Recognizing this gap, the current systematic review discusses the causative and protective nature of gut microbes in the context of HTN.

## Methodology

Initiated in June 2024, we conducted an electronic search for randomized or non-randomized controlled trials and registry studies in the PubMed and Cochrane databases. The following search strategy was used:

*((“Hypertension”[Mesh]) OR (high blood pressure)) AND (microbiome OR microbiota OR Gut microbiome OR microflora OR dysbiosis OR Probiotics OR Prebiotics OR Postbiotics)*

**Fig. 1:**
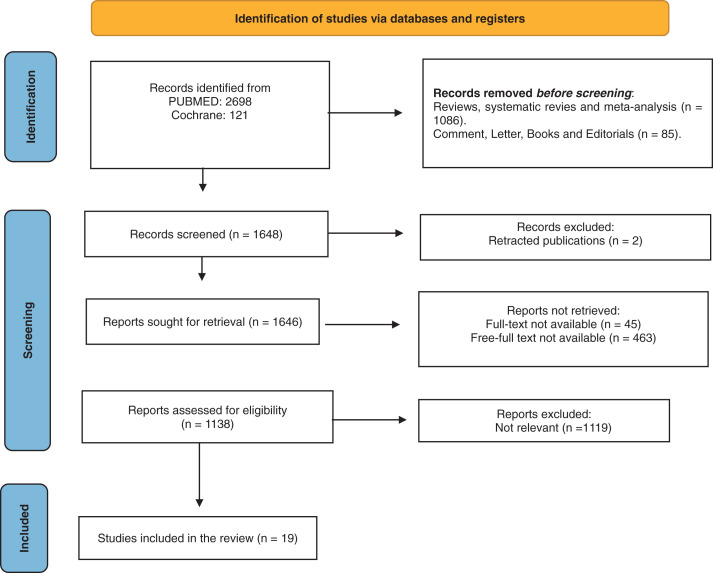
PRISMA flow diagram of systematic review methodology. PRISMA: Preferred Reporting Items for Systematic reviews and Meta-Analyses

A detailed PRISMA (Preferred Reporting Items for Systematic reviews and Meta-Analyses) flow diagram of the search approach is depicted in **Fig. 1**.

### Review

The current search identified a total of 19 studies to be included in the review (**Table 1**). The studies were categorized into 2 groups based on the influence of gut microbiota on HTN: those demonstrating a positive effect, a negative effect, and those with both.

**Table 1:** List of included studies

S. No	Author et al., year or study name	Study design	Study population/groups	Methodology/intervention	Results of the study
**Studies highlighting the potential of probiotics/gut microbiome in improving hypertension**					
1	Palmu et al., 2020^14)^	Registry study	829 finnish individuals Aged 25–74 years	BP measurement, stool collection, and 24-h urine sampling was performed. Shallow shotgun metagenome sequencing was performed to understand the gut microbiome profile.	In the study population, α- (within-sample) and β- (between-sample) diversities of taxonomic composition were strongly related to BP indexes (*P* <0.001). Mostly positive associations were found between BP indexes and 27 species of phylum Firmicutes (*P* <0.05). Mostly negative associations between 19 distinct *Lactobacillus* species and BP indexes (*P* <0.05). *Lactobacillus paracasei* was associated with lower mean arterial pressure (*P* <0.001) and lower dietary sodium intake (*P* <0.001).
2	Kang et al., 2023^15)^	Cross-sectional study	125 patients with primary grade-2 HTN 65 patients (BP not under control) 60 patients (BP under control)	16s rDNA sequencing of fecal intestinal flora was performed to analyze the differences in intestinal flora between the 2 groups of patients and to investigate the relationship between BP compliance and the presence of flora.	The intestinal flora of the 2 groups of patients differed in terms of the Firmicutes–Bacteroidetes ratio (F/B), α-diversity and β-diversity analysis results. *Streptococcus* and *Paraprevotella* in patients in the uncontrolled group were greater than that of the controlled group. *Akkermansia* and *Bifidobacterium* were higher in the controlled group. A logistic regression analysis of the difference factors found differences in ACE, F/B, *Streptococcus*, *Paraprevotella*, and *Akkermansia* in the 2 groups.
3	Lu et al., 2021^16)^	Cross-sectional study	128 individuals with HTN Divided into 2 groups based on the Montreal Cognitive Assessment: individuals with cognitive decline (HTNCI) and those without cognitive decline (HTNnCI)	Clinical and biological data. Gut microbiota composition estimation using16S ribosomal RNA sequencing, and the dominant species were identified by linear discriminant analysis.	The current study observes a higher abundance of TM7 and lower abundance of *Veillonella* and *Peptoniphilus* in the HTNCI group than in the HTNnCI group.
4	Susic et al., 2022^17)^	Cross-sectional study	18 mother–infant pairs with HTN	Differences in gut microbiota diversity and composition were observed between HP and NP women and infants.	HP women exhibited enriched gut microbiota with *Bifidobacterium* species and depleted *Barnesiella* species compared to NP women. HP infants showed lower gut microbiota diversity and were enriched with *Streptococcus infantis*, while being depleted in several beneficial bacterial strains compared to NP infants.
5	Tomsett et al., 2020^18)^	Cross-sectional study	22 women who were HP post-pregnancy 152 women who remained NP after pregnancy	Gut microbiota composition was examined using 16S rRNA gene amplicon sequencing. The gut permeability marker zonulin was measured in a subset.	Higher dietary fiber intake correlated with altered gut microbiota composition, characterized by an increased abundance of *Veillonella* and a decreased abundance of certain bacteria. However, this association was not observed in pregnancies with future HDP, suggesting altered gut barrier function in these cases compared to NP pregnancies.
6	Glazunova et al., 2022^19)^	Preclinical study	Milk fermented with several strains of traditional yogurt starters (*Lactobacillus delbrueckii* strains Lb100 and Lb200; *Lactococcus lactis* strains dlA, AM1, and MA1; *Streptococcus thermophilus* strains 159 and 16t), and 1 strain of non-conventional probiotic starter (*Lacticaseibacillus paracasei* ABK)	In vitro assessment: angiotensin-converting enzyme inhibition assay In vivo assessment: SHR animal model	Milk fermented with 2 strains (*Lb. delbrueckii* LB100 and *Lc. lactis* AM1) demonstrated significant anti-HP effect during both in vitro and in vivo experiments. Moreover, the milk fermented with *Lb. delbrueckii* Lb100 demonstrated significantly better fatty acid-related nutritional indexes and lowered total cholesterol in SHRs upon regular consumption.
7	Preez et al., 2020^20)^	Preclinical study	Male Wistar rats Protocol duration: 16 weeks	Group 1: Corn starch diet-fed rats (C) Group 2: C-rats supplemented with 5% *S. filiforme* for the last 8 weeks (CSF) Group 3: High-carbohydrate, high-fat diet-fed rats (H) Group 4: H-rats supplemented with 5% *S. filiforme* for the last 8 weeks (HSF)	H rats developed obesity, HTN, dyslipidemia, glucose intolerance, fatty liver, and increased left ventricular collagen deposition. *S. filiforme* supplementation decreased body weight, abdominal and liver fat, SBP, plasma total cholesterol concentrations, and plasma activities of alanine transaminase and aspartate transaminase. *S. filiforme* supplementation modulated gut microbiota without changing the Firmicutes to Bacteroidetes ratio. *S. filiforme* improved symptoms of high-carbohydrate, high-fat diet-induced metabolic syndrome in rats.
**Studies highlighting the role of gut microbiome involved in pathogenesis/worsening of hypertension**					
8	Chen et al., 2023^21)^	Cross-sectional study	Case: 95 periodontitis patients with HTN Control: 39 periodontitis patients without HTN	Saliva, subgingival plaques, and feces were collected for 16S rRNA gene sequencing or metagenomic analysis. C57BL/6J mice were pretreated with antibiotics to deplete gut microbiota and transplanted with human saliva by gavage.	Genera such as *Veillonella* were stably enriched in HP participants compared to controls. Saliva from HP individuals increased BP in control mice, implicating *Veillonella* and potentially other microorganisms in contributing to the progression of HTN.
9	Yan et al., 2017^13)^	Comparative study	Case: 60 patients with primary HTN Control: 60 age-, gender-, and weight-matched controls	Fecal samples were obtained, and the gut microbiome composition was estimated using whole-metagenome shotgun sequencing.	Higher prevalence of opportunistic pathogens such as *Klebsiella* spp. (*P* <0.05), *Streptococcus* spp. (*P* <0.01), and *Parabacteroides merdae* (*P* <0.01) in the gut microbiome of HP individuals. Beneficial bacteria, including *Roseburia* spp. and *Faecalibacterium prausnitzii*, were significantly higher in healthy controls (*P* <0.05).
10	Virwani et al., 2023^22)^	Cross-sectional study	241 participants 113 Male 128 Female	The following were estimated: GM-derived short-chain fatty acids GM characterized by shotgun sequencing, 24-h ambulatory BP	Bacteria such as *Ruminococcus gnavus* (*P* = 0.012), *Clostridium bolteae* (*P* = 0.038), and *Bacteroides ovatus* (*P* = 0.045) were found to be more abundant in HP women compared to NP women. *Dorea formicigenerans* was more abundant in the NP women.
11	Zou et al., 2022^23)^	Cross-sectional study	108 participants Groups: Pre-HTN (n = 38) HTN (n = 46) NT (n = 34)	Fecal and blood samples were collected, and the ITS transcribed spacer ribosomal RNA gene sequencing was performed. An immunoturbidimetric test was used to examine the serum levels of immunological LCs.	The relative abundance of *Malassezia* increased in the HTN group compared to that in the NT group. In the pre-HTN group, the relative abundance of *Malassezia* was positively associated with serum the concentration of LC. In the HTN group, the relative abundance of *Mortierella* was positively associated with the serum concentration of LC.
12	Hogue et al., 2024^24)^	Experimental study	30 African American collegiate athletes Groups: Normal BP HTN	16S rRNA gene sequencing was performed on stool samples to identify microbes at the genus level.	No significant differences in the α- and β-diversity. Significant correlations between SBP levels and the abundances of specific microbial genera, including *Adlercreutzia*, *Coprococcus, Granulicatella*, and *Veillonella* were observed.
13	Groot et al., 2020^25)^	Registry study	422417 participant details obtained from the UK Biobank	Comprehensive Mendelian randomization analysis	The abundance of *Ruminococcus flavefaciens* was observed to influence the risk of HTN
14	Lan et al., 2022^26)^	Prospective cohort study	1242 participants – surveyed GM estimation: 113 Han people (55 with HTN and 58 without HTN) and 40 Yugur people (23 with HTN and 17 without HTN)	16S rRNA gene sequencing for determining the gut microbiome composition	The current study observed a 1.8 times higher prevalence of HTN (38.2–43.3%) than the average in China (23.2%), under a special high-calorie diet based on wheat, cattle, mutton, and animal offal. The high-calorie diet led to gut microbial dysbiosis characterized by a marked depletion of *Lachnospiraceae* genera and was associated with pathogenesis of HTN.
15	Luo er al., 2023^27)^	Preclinical study	44 subjects with elevated arterial stiffness and 45 age- and sex-matched normal controls	Angiotensin II (Ang II)-induced and humanized mouse models were employed to evaluate the protective effect of *Flavonifractor plautii* (*F. plautii*) and its main effector cis-aconitic acid.	Metagenomic sequencing revealed a significantly high abundance and centrality of *F. plautii* in normal controls, which was absent in the microbial community of subjects with elevated arterial stiffness. Replenishment with *F. plautii* improved elastic fiber network and reversed increased pulse wave velocity through a myriad of mechanisms.
16	Luo et al., 2022^28)^	Registry study	18340 individuals	Two-sample Mendelian randomization analysis was performed using MR-Egger, inverse variance weighted (IVW), MR-PRESSO, maximum likelihood, and weighted median.	For every unit increase in *Shigella* concentration, the relative risk increased by 38.1% for myocarditis and by 13.3% for hypertrophic cardiomyopathy. For every unit increase in *Candida* concentration, the relative risk of chronic kidney disease increased by 7.1%. For every unit increase in betaine, the relative risk of heart failure and myocardial infarction increased by 1.4% and 1.7%, respectively. All of these conditions have been associated with HTN.
17	Han et al., 2018^29)^	Registry study	196 human faecal samples	The viral composition and bacterial composition of the samples were identified, and their associations with different HP states were determined.	32 significant viral biomarkers were identified for distinguishing healthy, pre-HP, and HP individuals.
18	Toral et al., 2019^30)^	Experimental study	NP WKY and SHR Group A: WKY with WKY microbiota (W–W) Group B: WKY with SHR microbiota (W–S) Group C: SHR with SHR microbiota (S–S) Group D: SHR with WKY microbiota (S–W)	Recipient NP WKY and SHRs were orally gavaged with donor fecal contents from SHR and WKY.	The results demonstrated that FMT from WKY rats to SHR significantly reduced basal SBP and diastolic BP, which was associated with decreased markers of neuroinflammation and sympathetic nervous system activity in SHR. Conversely, FMT from SHR to WKY rats led to increased BP and inflammatory markers in WKY rats. Correlation analyses further identified specific microbiota, such as *Blautia* and *Odoribacter*, as being negatively correlated with high SBP in SHR.
19	Lv et al., 2023^31)^	Prospective Cohort Study	87 HTN subjects and 45 controls	16S rRNA gene sequencing for determining the gut microbiome composition	The enrichment of *Leuconostocaceae*, *Weissella*, and *Weissella cibaria* in control females suggests that these microbes are more abundant in individuals without HTN. Additionally, the positive correlation of functional classifiers such as Cellular Processes, Human Diseases, Signal Transduction, and Two-component System (all *P* <0.05) with SBP indicates that increased activity or prevalence of these processes is associated with higher BP.

BP: blood pressure; GM: gut microbiome; HP: hypertensive; HTN: hypertension; LC: light chain; MR: Mendelian Randomization Pleiotropy RESidual Sum and Outlier; NP: normotensive; NT: normal tension; PRISMA: Preferred Reporting Items for Systematic reviews and Meta-Analyses; rRNA: ribosomal RNA; SBP: systolic blood pressure; SHR: spontaneously hypertensive rat; WKY: Wistar–Kyoto

### Microorganisms in the gut associated with improvement in HTN

Recent studies have shed light on the intricate relationship between gut microbiota composition and HTN, highlighting potential avenues for therapeutic intervention. Palmu et al., involving 6953 Finnish participants, highlighted weak associations between overall gut taxonomic composition and blood pressure (BP). They found notable changes in several microbial genera in individuals with HTN, with significant associations observed between BP indexes and 45 microbial genera, predominantly from the Firmicutes phylum. Notably, specific *Lactobacillus* species, such as *Lactobacillus paracasei*, exhibited strong negative associations with BP, emphasizing potential dietary sodium intake implications and suggesting avenues for further experimental exploration.^14)^ Kang et al. conducted a study involving 125 grade-2 HTN patients, revealing substantial differences in gut microbiota composition between those with well-controlled and poorly controlled BP. Microbial markers such as ACE, *Streptococcus*, and *Akkermansia* were identified as potential predictors of long-term BP control. This study highlights the therapeutic potential of targeting specific gut microbial markers to manage HTN effectively.^15)^ Lu et al. expanded on these insights by exploring the association between gut microbiota and cognitive impairment in hypertensive (HP) patients. Their observational study of 128 individuals identified *Veillonella* species as potential biomarkers linked to cognitive decline in HP populations, indicating broader implications of gut microbial composition beyond BP regulation.^16)^

In another experimental study by Susic et al. involving 18 mother–infant pairs, differences in gut microbiota diversity and composition were observed between HP and normotensive (NP) women and infants. HP women exhibited enriched gut microbiota with *Bifidobacterium* species and depleted *Barnesiella* species compared to NP women. HP infants showed lower gut microbiota diversity and were enriched with *Streptococcus infantis*, while being depleted in several beneficial bacterial strains compared to NP infants. These findings underscore the developmental implications of maternal HTN on infant gut microbiota and potential health outcomes.^17)^

Moreover, Tomsett et al. conducted a cross-sectional study investigating dietary fiber intake and its association with gut microbiota composition in women at risk of HP disorders of pregnancy (HDP). They found that higher dietary fiber intake correlated with altered gut microbiota composition, characterized by an increased abundance of *Veillonella* and a decreased abundance of certain other bacteria. However, this association was not observed in pregnancies with future HDP, suggesting altered gut barrier function in these cases compared to NP pregnancies. The study investigated dietary influences on gut microbiota in pregnant women at risk for HP disorders, observing altered microbial communities with varying fiber intake. Although the study did not find improved gut barrier function with higher fiber intake, it noted significant changes in microbial composition, including increased *Veillonella* abundance, suggesting potential dietary strategies to modulate gut health in HP conditions.^18)^

Further highlighting the therapeutic potential, interventions such as fermented milk products enriched with specific bacterial strains^19)^ and dietary changes (e.g., carrageenans from *Sarconema filiforme* attenuating symptoms of diet-induced metabolic syndrome in rats) have shown promising anti-HP effects through modulation of gut microbiota composition and function.^20)^ These studies emphasize the role of microbial metabolites, such as short-chain fatty acids (SCFAs) and bile acids, in mediating cardiovascular health outcomes through their effects on systemic inflammation, oxidative stress, and vascular function.

### Microorganisms in the gut associated with worsening HTN

Several studies have identified specific microorganisms within the gut microbiome that may exacerbate HTN by altering microbial composition and metabolic activities. For instance, Chen et al. conducted a cross-sectional study followed by a 6-month follow-up to investigate the roles of oral and gut microbiota in HTN. They found that certain genera, including *Veillonella*, were stably enriched in HP participants compared to controls. Saliva from HP individuals increased BP in HP mice, implicating *Veillonella* and potentially other microorganisms in contributing to the progression of HTN.^21)^

Yan et al. conducted a comparative study involving 60 patients with primary HTN and 60 gender-, age-, and body weight-matched healthy controls. Their findings revealed a higher prevalence of opportunistic pathogens such as *Klebsiella* spp. (*P* <0.05), *Streptococcus* spp. (*P* <0.01), and *Parabacteroides merdae* (*P* <0.01) in the gut microbiome of HP individuals. Conversely, beneficial bacteria known for producing SCFAs, such as *Roseburia* spp. and *Faecalibacterium prausnitzii*, were significantly higher in healthy controls (*P* <0.05). These differences highlight specific microbial alterations associated with HTN and suggest a role for dysbiosis in HTN pathogenesis.^13)^

In another study by Virwani et al., which focused on a cross-sectional analysis of 241 Hong Kong Chinese participants (113 men and 128 women), significant differences in gut microbiome composition were observed between HP and NP women. Bacteria such as *Ruminococcus gnavus* (*P* = 0.012), *Clostridium bolteae* (*P* = 0.038), and *Bacteroides ovatus* (*P* = 0.045) were found to be more abundant in HP women, potentially linking these microbial profiles to HTN development through unknown mechanisms.^22)^

Furthermore, Zou et al. investigated fungal dysbiosis in individuals with pre-HTN, HTN, and normal BP. They identified dysregulation in fungal taxa such as *Malassezia* (*P* <0.01) and *Mortierella* (*P* <0.05), with significant differences between HP and NP groups. This dysbiosis correlated with alterations in serum indicators, suggesting potential fungal biomarkers that may be indicative of HTN risk.^23)^

Hogue et al. conducted an experimental study involving 30 African American collegiate athletes, categorized into normal BP (systolic BP (SBP) ≤130 mmHg) and HTN groups (SBP ≥130 mmHg). They found significant correlations between SBP levels and the abundances of specific microbial genera, including *Adlercreutzia, Coprococcus, Granulicatella*, and *Veillonella*. This suggests that variations in these microbial populations could potentially influence SBP levels among athletes.^24)^

Meanwhile, Groot et al. performed a comprehensive Mendelian randomization analysis using data from 422417 participants in the UK Biobank. Their findings implicated gut microbiota, particularly *Ruminococcus flavefaciens*, in influencing HTN risk, underscoring the significant role of microbial factors in cardiovascular health.^25)^ Furthermore, the study by Lan et al. examined 1242 Yugur and Han individuals and highlighted that HTN, compounded by a high-calorie diet, led to gut microbial dysbiosis characterized by a marked depletion of *Lachnospiraceae* genera. This microbial imbalance was associated with HTN pathogenesis, indicating a link between dietary factors, gut microbiota composition, and cardiovascular outcomes.^26)^ Additionally, Luo et al. conducted a comparative study focusing on participants with elevated arterial stiffness, a precursor to HTN. They identified *Flavonifractor plautii* as a key microbial species potentially influencing arterial stiffness, thereby impacting cardiovascular health.^27)^

Using Mendelian randomization, a study by Luo et al. examined various conditions, including HTN, highlighting specific gut bacteria and metabolites associated with cardiovascular diseases. This included findings on microbial species such as *Candida, Shigella*, and *Campylobacter*, which were linked to increased risks of myocarditis and hypertrophic cardiomyopathy, illustrating diverse microbial influences on cardiovascular health.^28)^

A notable observational study conducted by Han et al., involving 196 fecal samples, investigated the viral composition and its associations with different HTN states. The study identified 32 significant viral biomarkers, demonstrating superior resolution and discriminatory power compared to bacterial markers in distinguishing healthy, pre-HTN, and HTN samples. The findings revealed increasingly pervasive virus-bacteria linkages from healthy individuals to those with pre-HTN and HTN, suggesting a progressive alteration in the gut microbiome’s composition and interactions as HTN develops. These results highlight the virome’s complexity and potential role in HP conditions, advocating for further exploration into the gut virome’s role in HTN.^29)^

An experimental study investigated the critical role of gut microbiota interactions with the sympathetic nervous system in regulating BP using fecal microbiota transplantation (FMT) in animal models, specifically NP Wistar–Kyoto (WKY) rats and spontaneously HP rats (SHR). The results demonstrated that FMT from WKY rats to SHR significantly reduced basal systolic and diastolic BP, which was associated with decreased markers of neuroinflammation and sympathetic nervous system activity in SHR. Conversely, FMT from SHR to WKY rats led to increased BP and inflammatory markers in WKY rats. Correlation analyses further identified specific microbiota, such as *Blautia* and *Odoribacter*, as being negatively correlated with high SBP in SHR.^30)^

A study among 87 HTN subjects and 45 control individuals residing in Northwestern China revealed that women without HTN had a higher abundance of *Leuconostocaceae* (a family), *Weissella* (a genus), and *Weissella cibaria* (a species) than women with HTN.^31)^ A positive correlation of functional classifiers such as “Cellular Processes” (*P* <0.05), “Human Diseases” (*P* <0.05) and “Signal transduction” (*P* <0.05), and “Two-component system” (*P* <0.05) with SBP suggests that as these processes or systems become more active or prevalent, BP tends to increase. Therefore, such functional classifiers based on biological processes may be used to predict or diagnose HTN in women.^31)^ *Weissella* species, particularly *W. cibaria*, have been noted for their probiotic properties, such as biofilm inhibition, anti-inflammatory effects, and potential benefits for oral health. This highlights the possible role of gut microbiome changes in HTN, especially in women.^32)^

These studies collectively highlight the multifaceted role of gut microbiota in both exacerbating and mitigating HTN risk through intricate microbial interactions and metabolic pathways. They pave the way for targeted therapeutic interventions and personalized approaches leveraging the gut microbiome to manage HTN and related cardiovascular conditions effectively.

## Conclusion

This systematic review highlights the possible roles of various gut microbiota in HTN. Probiotic species/genera such as *L. paracasei*, *Akkermansia*, and *Veillonella* appear to be involved in the regulation of BP. HP subjects show an increase in *Klebsiella* sp., *Streptococcus* sp., and *P. merdae*, which are known to be pro-inflammatory and dysbiotic. Moreover, the fungal groups *Malassezia* and *Mortierella* have also been shown to be involved in HTN pathogenesis. These microbial signatures form a foundation for microbiota-targeted therapies, such as probiotics, prebiotics, and nutritional changes as potential interventions for HTN control. More mechanistic research and large-scale clinical trials are required to determine causal associations and to confirm these strategies, with future efforts involving personalized interventions based on an individual’s gut microbiota composition for enhanced treatment efficacy.
